# Diverting ileostomy for hemorrhagic proctitis after cardiovascular surgery in a patient with prior radiotherapy: a case report

**DOI:** 10.1097/RC9.0000000000001047

**Published:** 2026-09-21

**Authors:** Kenichiro Yambe, Shingo Tsujinaka, Tomoya Miura, Shunsuke Kawamoto, Chikashi Shibata, Yu Katayose

**Affiliations:** a Division of Hepato-Biliary and Pancreatic Surgery, Tohoku Medical and Pharmaceutical University, Sendai, Japan; b Division of Gastroenterologic Surgery, Tohoku Medical and Pharmaceutical University, Sendai, Japan; c Division of Cardiovascular Surgery, Tohoku Medical and Pharmaceutical University, Sendai, Japan

**Keywords:** acute aortic dissection, distal pancreatectomy, diverting ileostomy, hemorrhagic proctitis, radiotherapy

## Abstract

**Introduction and importance:**

Hemorrhagic proctitis after cardiovascular surgery is rare, and no established strategy exists when bleeding is refractory but the rectal injury appears reversible.

**Presentation of case:**

A 74-year-old man with prior pelvic radiotherapy for prostate cancer underwent distal pancreatectomy for an intraductal papillary mucinous neoplasm. On postoperative day (POD) 2, he developed Stanford type A acute aortic dissection with cardiac tamponade and underwent emergency total arch replacement with open stent-graft insertion. Abdominal pain developed on POD 7, followed by massive hematochezia on POD 12. Colonoscopy revealed a circumferential rectal ulcer with diffuse mucosal oozing, and biopsy demonstrated mucosal necrosis without malignancy or viral inclusions. Endoscopy and computed tomography, a normal lactate level (1.2 mmol/L), and the absence of peritoneal signs suggested nontransmural, potentially reversible injury. Bleeding persisted despite bowel rest, transfusion, packing, topical thrombin, and endoscopic therapy. Laparoscopic diverting loop ileostomy was performed on POD 17 to control bleeding while preserving the rectum. The ulcer improved, and he was transferred for rehabilitation on POD 47. Complete ulcer healing was confirmed, and the ileostomy was closed 2 years later. At the latest follow-up, he remained ambulatory without recurrent proctitis, tumor recurrence, or cardiovascular events.

**Clinical discussion:**

This case combines acute aortic dissection immediately after abdominal surgery, a hemorrhagic rectal ulcer possibly reflecting perioperative hypoperfusion superimposed on radiation-related vulnerability rather than watershed ischemia, and rectal preservation by diversion rather than embolization, colostomy, or resection.

**Conclusion:**

A diverting ileostomy may be a bowel-preserving salvage option for refractory hemorrhagic proctitis when the rectal injury appears reversible.

## Introduction

Acute ischemic proctitis is rare, accounting for less than 5% of rectal lesions, but may cause massive bleeding requiring endoscopic, radiological, or surgical treatment^[^1^]^. Cardiovascular complications occur in approximately 0.7% of major abdominal surgeries^[^2^]^, whereas gastrointestinal complications after cardiac surgery occur in 0.5%–5.5% of cases and include intestinal ischemia, which carries high mortality^[^3,4^]^. Prior pelvic radiotherapy may produce chronic microvascular and mucosal injury that reduces rectal tolerance to subsequent hypoperfusion. However, no established strategy exists for refractory ischemic and hemorrhagic proctitis in an irradiated rectum when bleeding is life-threatening, but imaging and endoscopy suggest nontransmural injury.

HIGHLIGHTS

- An acute aortic dissection occurred early after distal pancreatectomy.
- Refractory hemorrhagic proctitis developed after cardiovascular surgery.
- Prior radiotherapy and hypoperfusion may synergistically cause rectal ulceration.
- An ileostomy may be a bowel-preserving option for reversible hemorrhagic proctitis.

Here, we present a rare case of refractory hemorrhagic proctitis with a circumferential rectal ulcer after a postoperative aortic dissection, successfully treated with a diverting ileostomy as a bowel-preserving strategy. This case is reported in accordance with the Surgical Case Report (SCARE) guidelines^[^5^]^.

## Presentation of case

The clinical course is summarized in Table 1.

**Table 1:** Timeline of the clinical course.

POD	Event
0	Open spleen-preserving distal pancreatectomy for intraductal papillary mucinous neoplasm
2	Stanford type A acute aortic dissection with cardiac tamponade; emergency total arch replacement with open stent-graft insertion
6	Computed tomography confirming patent major aortic branches (superior mesenteric artery, inferior mesenteric artery, and bilateral internal iliac arteries)
7	Abdominal pain; Computed tomography showing rectal wall thickening
12	Massive hematochezia; colonoscopy showing a circumferential rectal ulcer; biopsy showing mucosal necrosis
13–15	Mild hematochezia; no transfusion; stable hemoglobin
16	Persistent diffuse oozing; 6 units of red blood cells transfused; rectal gauze packing
17	Recurrent massive hematochezia; topical thrombin ineffective; laparoscopic double-barreled loop ileostomy (Clavien–Dindo grade IIIb)
26	Endoscopy showing ulcer improvement
39	Computed tomography showing resolution of rectal wall thickening
40	Colonoscopy showing epithelial regeneration
47	Transfer for rehabilitation
2 years	Colonoscopy confirming healing; ileostomy closure

### Present illness and past medical history

A 74-year-old man underwent open, spleen-preserving distal pancreatectomy for an intraductal papillary mucinous neoplasm of the pancreatic tail. His medical history included hypertension, hyperuricemia, distal gastrectomy for a gastric ulcer, and prostate cancer treated 3 years earlier with three-dimensional conformal radiotherapy (74 Gy in 37 fractions); the rectum was adjacent to the radiation field. He had no prior rectal bleeding or symptoms of chronic radiation proctitis. His preoperative medications were olopatadine, candesartan, glycyrrhizic acid, oxybuprocaine, allopurinol, esomeprazole, and clotiazepam; he was not receiving antiplatelet or anticoagulant therapy. He was a former smoker (20 cigarettes per day from ages 20 to 40), did not drink alcohol, and was independent in activities of daily living. Preoperative computed tomography (CT) showed mild ascending-aortic dilatation (41 mm) without a penetrating atherosclerotic ulcer, significant atheroma, or substantial calcification.

### Acute aortic dissection

Unless otherwise stated, postoperative days (PODs) are counted from distal pancreatectomy (POD 0). On POD 2, sudden chest and back pain developed, with a blood pressure of 82/45 mmHg and a heart rate of 110/min. An emergency CT scan showed a retrograde DeBakey type IIIc, Stanford type A, dissection with cardiac tamponade (Fig. 1A); the celiac, superior mesenteric artery (SMA), and inferior mesenteric artery (IMA) arose from the true lumen, whereas the left renal artery arose from the false lumen. After pericardiocentesis and hospital transfer, the blood pressure was 62/35 mmHg, the heart rate was 99/min, and the lactate level was 8.1 mmol/L.

**Figure 1.**
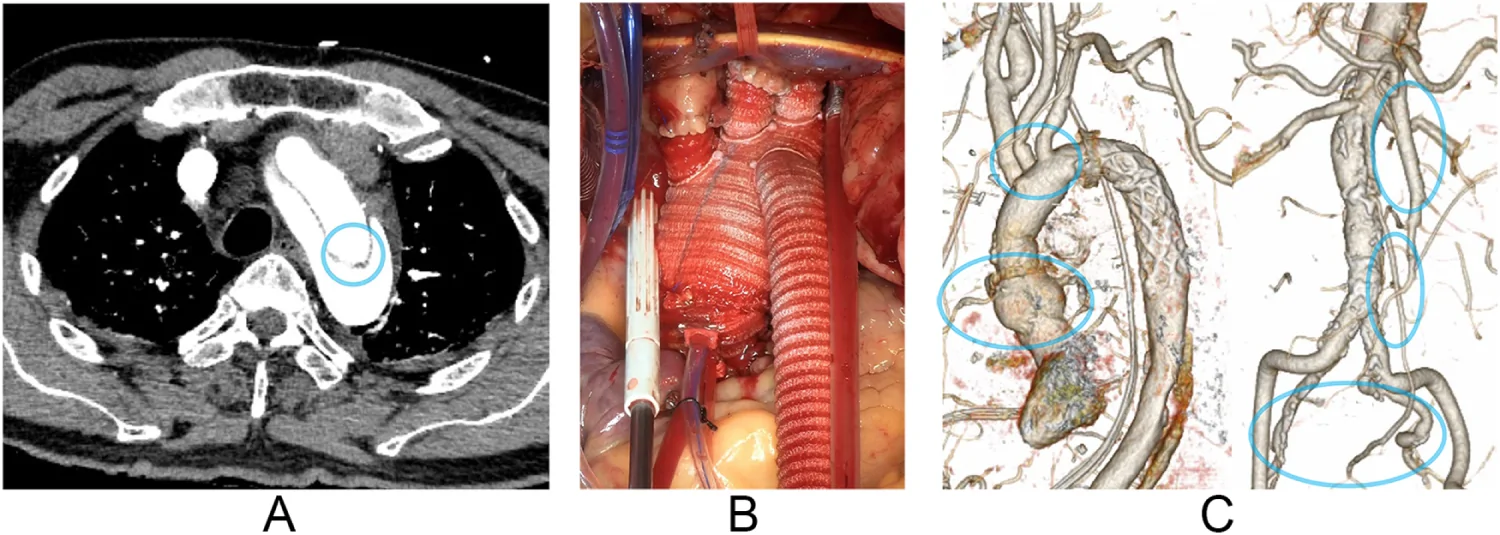
Total arch replacement and open stent-graft insertion. (A) Contrast-enhanced computed tomography at the onset of the aortic dissection (circle, entry site). (B) Post-vascular anastomosis. (C) Postoperative three-dimensional contrast-enhanced computed tomography showing patent major aortic branches, the superior mesenteric artery, the inferior mesenteric artery, and the bilateral internal iliac arteries (circles).

Emergency total arch replacement with open stent-graft insertion was performed (Fig. 1B); cardiopulmonary bypass lasted 287 minutes, selective cerebral perfusion 133 minutes, circulatory arrest 68 minutes, and the minimum temperature was 24.4°C. Blood loss was 1601 mL, requiring 6 units of red blood cells (RBCs), 20 units of fresh-frozen plasma, and 20 units of platelet concentrates. No postoperative antiplatelet or anticoagulant therapy was given. CT on POD 6 confirmed patency of the major aortic branches and the SMA, IMA, and bilateral internal iliac arteries (Fig. 1C).

### Hematochezia due to rectal ulcer

On POD 7, sudden abdominal pain developed, and CT revealed rectal wall thickening (Fig. 2A). Massive hematochezia began on POD 12; colonoscopy demonstrated a circumferential friable ulcer (Fig. 2B), and biopsy showed extensive mucosal necrosis without malignancy or viral inclusions. Hematochezia remained mild with stable hemoglobin and no transfusions on PODs 13–15, but persistent oozing on POD 16 required 6 units of RBCs and rectal gauze packing. Baseline hemoglobin was 9.1 g/dL, the nadir during PODs 12-17 was 6.5 g/dL, and 22 units of RBCs were transfused cumulatively. Transfusion was guided by active bleeding, hemoglobin decline, and hemodynamic status. Transient prolongation of activated partial thromboplastin time occurred when continuous hemodiafiltration began. Hypofibrinogenemia on POD 24 was treated with 8 units of fresh-frozen plasma; no other major coagulation abnormalities occurred.

**Figure 2.**
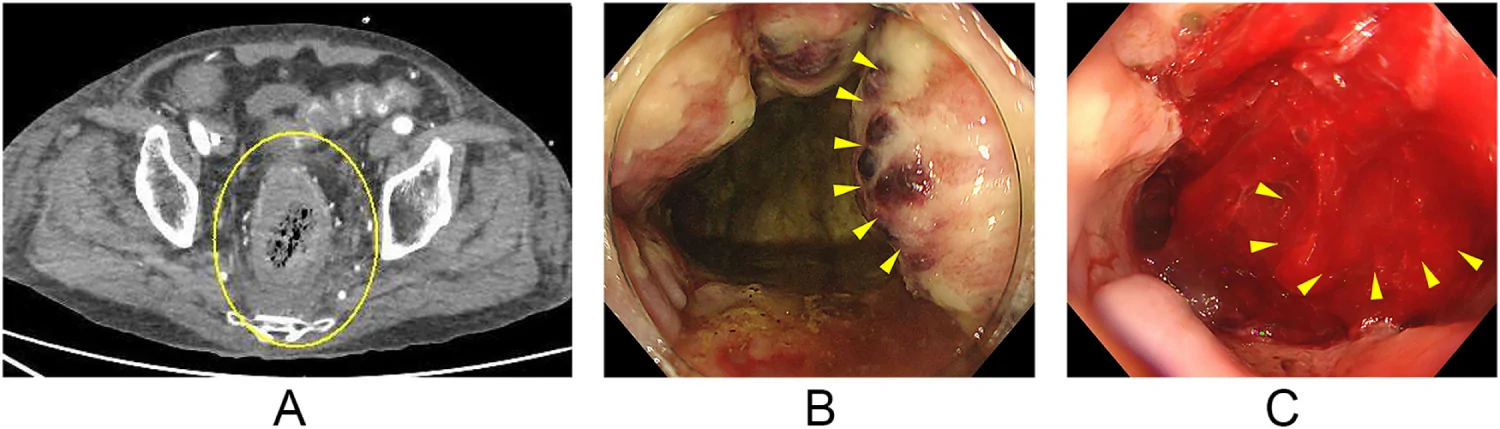
Rectal ulcer before diverting loop ileostomy. (A) POD 7: Contrast-enhanced computed tomography shows rectal wall thickening with contrast enhancement (circle). (B) POD 12: Colonoscopy at the onset of hematochezia shows a circumferential rectal ulcer with adherent necrotic material (arrowheads). (C) POD 17: Second-look colonoscopy shows diffuse mucosal bleeding and intraluminal coagula (arrowheads).

On POD 17, massive bleeding recurred as diffuse oozing over the ulcer (Fig. 2C). Conservative management included fasting, parenteral nutrition, meropenem and vancomycin for suspected bacterial translocation, transfusion, rectal packing, and topical thrombin; bleeding persisted, and clips or cautery were unsuitable for the diffuse lesion. Repeated transfusions and continuous vasopressors were required. The ulcer was confined to the irradiated rectum rather than to a typical watershed area.

Because endoscopy and CT suggested a non-full-thickness injury, the recent major cardiovascular operation limited physiological reserve, and permanent loss of a nonmalignant rectum was undesirable, a laparoscopic double-barreled loop ileostomy was performed on POD 17. Diversion was intended to remove fecal stimulation, facilitate mucosal healing, and preserve rectal perfusion and sphincter function, with less operative stress than resection. Its principal risk was persistent hemorrhage, requiring conversion to Hartmann’s procedure or abdominoperineal resection. The unplanned reoperation was classified as Clavien–Dindo grade IIIb.

Endoscopic findings on POD 26 showed improvement in the ulcer (Fig. 3A), contrast-enhanced CT on POD 39 demonstrated resolution of rectal wall thickening (Fig. 3B), and colonoscopy on POD 40 confirmed progressive healing and epithelial regeneration (Fig. 3C). Nutrition was advanced from parenteral to enteral.

**Figure 3.**
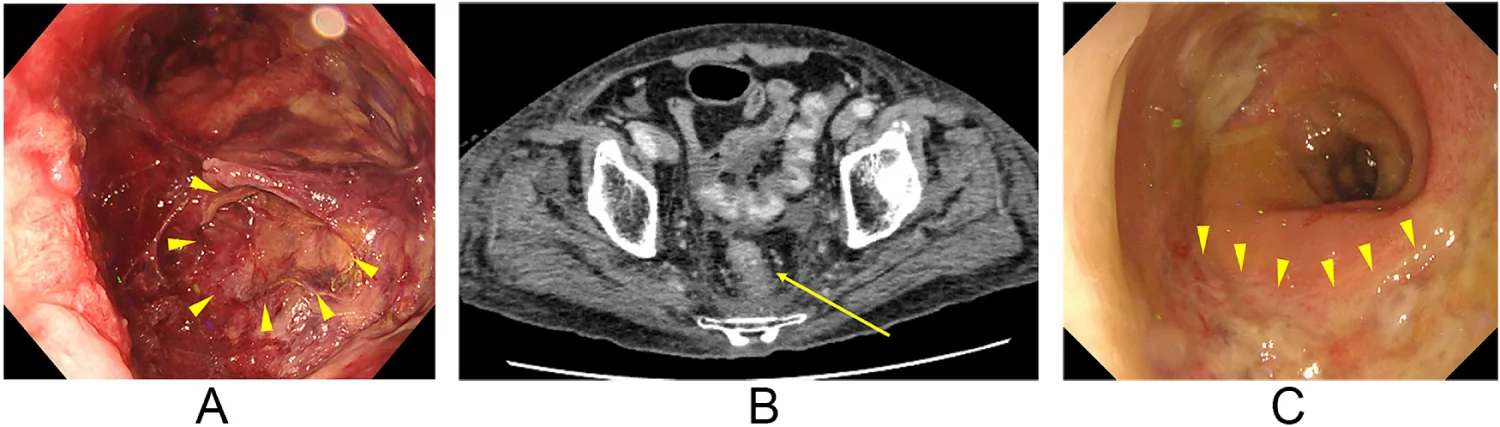
Rectal ulcer after a diverting loop ileostomy. (A) POD 26: Colonoscopy showing partial epithelial regeneration (arrowheads). (B) POD 39: Contrast-enhanced computed tomography showing resolution of rectal wall thickening (arrow). (C) POD 40: Colonoscopy showing regenerative epithelium (arrowheads).

### Clinical course after diverting ileostomy

The patient was transferred for rehabilitation on POD 47. Complete healing without stenosis was confirmed, and an ileostomy was performed approximately 2 years later. At the latest follow-up, he remained ambulatory without recurrent proctitis, bowel dysfunction, tumor recurrence, or cardiovascular events.

## Discussion

### Pathogenesis and diagnostic reasoning

To our knowledge, no report describes the complete sequence of acute type A dissection immediately after abdominal surgery, refractory hemorrhagic proctitis after cardiovascular surgery, and bowel-preserving diversion. Ischemic proctitis after cardiac surgery and rectal injury from shock or radiotherapy have been reported separately^[^1,3,4,6^]^. Radiotherapy causes endothelial injury, fibrosis, and chronic inflammation^[^6^]^; therefore, profound shock, prolonged cardiopulmonary bypass, circulatory arrest, hypothermia, and vasopressor exposure may have aggravated a previously vulnerable rectum. Although postoperative CT confirmed patent mesenteric and internal iliac arteries, macroscopic arterial patency does not exclude transient microcirculatory hypoperfusion. The absence of preceding proctitis and confinement to the irradiated segment support this hypothesis but do not establish causation.

### Assessment of reversibility and differential diagnosis

Reversibility was judged from concordant findings available before diversion: endoscopy showed ulceration with edematous but viable mucosa and no exposed muscularis or gangrenous discoloration; CT showed no pneumatosis, non-enhancement, abscess, or free gas; there were no peritoneal signs or progressive sepsis, hemodynamics remained responsive to resuscitation, and lactate was 1.2 mmol/L. Serial studies subsequently demonstrated regeneration, corroborating rather than defining the initial assessment. No validated severity score exists for ischemic proctitis, so these criteria were adapted from distinctions between reversible and gangrenous ischemic colitis^[^7^]^.

Classic ischemic colitis was less likely because the rectum has dual arterial supply from the inferior mesenteric and internal iliac arteries, and the lesion lay outside the usual watershed areas^[^1,7^]^. Non-occlusive mesenteric ischemia was less likely because the injury was localized rather than diffuse in the small bowel or right colon. Chronic radiation proctitis alone did not explain the abrupt onset after shock in a previously asymptomatic patient. Infectious colitis, including *Clostridioides difficile* and cytomegalovirus, was less likely because the lesion was confined to the rectum and biopsy showed no viral inclusions. We therefore diagnosed acute ischemic hemorrhagic proctitis superimposed on radiation-related vulnerability.

### Management options and rationale for fecal diversion

The circumferential oozing provided no focal target for endoscopic clipping, cautery, or selective arterial embolization, and topical thrombin failed. Moreover, embolizing an irradiated, hypoperfused rectum risked converting mucosal injury to transmural necrosis. Resection is appropriate for gangrene or otherwise uncontrollable bleeding^[^1^]^ and would have provided definitive source control, but it would also have imposed substantially greater stress soon after total arch replacement and permanently sacrificed a nonmalignant rectum. Diversion instead reduced fecal trauma while preserving perfusion and leaving resection available if bleeding continued.

Evidence for diversion is derived mainly from chronic radiation injury. Staged ileostomy achieved an 81% closure rate with a 19% complication rate in 21 patients^[^8^]^, and diverting colostomy achieved an 88% response at 12–24 months^[^9^]^. Although colostomy is also effective, these data are not directly equivalent to acute postoperative ischemic injury. In this patient, loop ileostomy avoided further manipulation of the potentially hypoperfused colon, limited operative burden, and facilitated later reversal^[^10^]^.

### Take-away lessons

Three clinical lessons emerge. First, postoperative chest or back pain with hemodynamic deterioration warrants urgent evaluation for aortic dissection, even without overt preoperative intimal disease. Second, an irradiated rectum may be susceptible to profound perioperative hypoperfusion and acute diffuse hemorrhagic ulceration. Third, when bleeding persists but multimodal assessment suggests viable, nontransmural tissue, temporary diversion may preserve the rectum and facilitate later stoma closure.

### Limitations

This single case cannot establish the effectiveness or optimal timing of diversion, and evidence supporting ileostomy is derived mainly from cases of chronic radiation injury. No validated severity score exists for ischemic proctitis; our assessment was extrapolated from ischemic-colitis criteria and depended partly on clinical judgment. The proposed radiotherapy-hypoperfusion interaction remains hypothetical because full-thickness histopathological confirmation, direct perfusion assessment, and comparative cases were unavailable. Accordingly, neither causality nor superiority over embolization, colostomy, or resection can be inferred.

## Conclusions

Diverting loop ileostomy may provide bowel-preserving salvage for refractory hemorrhagic proctitis after cardiovascular surgery when endoscopic, radiological, laboratory, and clinical findings indicate nontransmural, potentially reversible injury. Persistent bleeding or evidence of necrosis should nevertheless prompt definitive intervention.

## Data Availability

The data that support the findings of this study are available from the corresponding author upon reasonable request.
